# Bromeliads going batty: pollinator partitioning among sympatric chiropterophilous Bromeliaceae

**DOI:** 10.1093/aobpla/plz014

**Published:** 2019-03-12

**Authors:** Pedro Adrián Aguilar-Rodríguez, Marco Tschapka, José G García-Franco, Thorsten Krömer, M Cristina MacSwiney G

**Affiliations:** 1 Centro de Investigaciones Tropicales, Universidad Veracruzana, José María Morelos, Col. Centro, C.P. Xalapa, Veracruz, México; 2 Department of Zoology, Tel Aviv University, Tel Aviv, Israel; 3 Institute of Evolutionary Ecology and Conservation Genomics, University of Ulm, Albert Einstein Allee 11, D Ulm, Germany; 4 Smithsonian Tropical Research Institute, Balboa Ancón, Apartado, Panamá, Republica de Panamáa; 5 Red de Ecología Funcional, Instituto de Ecología, A.C., Carretera Antigua a Coatepec No. 351, El Haya, C.P. Xalapa, Veracruz, México

**Keywords:** *Anoura*, Bromeliaceae, chiropterophily, *Glossophaga*, humid montane forest, Mexico, *Pitcairnia*, pollinator effectiveness, *Pseudalcantarea*, *Werauhia*

## Abstract

Pollinators can be a limited resource and natural selection should favour differences in phenotypic characteristics to reduce competition among plants. Bats are important pollinators of many Neotropical plants, including the Bromeliaceae; however, the pre-pollination mechanisms for isolation among sympatric bat-pollinated bromeliads are unknown. Here, we studied the mechanisms for reproductive segregation between *Pitcairnia recurvata*, *Pseudalcantarea viridiflora*, *Werauhia noctiflorens* and *W. nutans*. The study was conducted at Los Tuxtlas Biosphere Reserve, in Veracruz, Mexico We carried out *ex situ* and *in situ* manual pollination treatments to determine the breeding system by assessing fruiting and seedling success and sampled bat visitors using mist-nets and infrared cameras. We determined the nocturnal nectar production pattern, estimating the energetic content of this reward. All four bromeliads are self-compatible, but only *P. recurvata* appears to require pollinators, because the physical separation between anthers and stigma prevents self-pollination, it is xenogamous and presents a strictly nocturnal anthesis. The bats *Anoura geoffroyi*, *Glossophaga soricina* and *Hylonycteris underwoodi* are probable pollinators of three of the studied bromeliads. We did not record any animal visiting the fourth species. The flowering season of each species is staggered throughout the year, with minimal overlap, and the floral morphology segregates the locations on the body of the bat where the pollen is deposited. The most abundant nectar per flower is provided by *P. viridiflora*, but *P. recurvata* offers the best reward per hectare, considering the density of flowering plants. Staggered flowering, different pollen deposition sites on the body of the pollinator and differences in the reward offered may have evolved to reduce the competitive costs of sharing pollinators while providing a constant supply of food to maintain a stable nectarivorous bat community.

## Teaser

Pre-pollination mechanisms for isolation among sympatric bat-pollinated plants are largely unknown. We investigated the mechanisms for reproductive segregation between four sympatric chiropterophilous bromeliads in Veracruz, Mexico. With *ex situ* and *in situ* manual pollination treatments we determined the breeding system by assessing fruiting and seedling success and sampled bat visitors using mist-nets and infrared cameras. Bromeliads showed staggered flowering, different pollen deposition sites on the body of the pollinator and differences in the reward offered. These traits may have evolved to reduce the competitive costs of sharing pollinators while providing a constant supply of food for the nectarivorous bat community.

## Introduction

Zoophilous pollination has been important for the evolution and diversification of angiosperms (Hu *et al.* 2008), with nearly 90 % of the extant angiosperms presenting this form of pollination (Ollerton *et al.* 2011). However, many plant species use a variety of different animals as pollinators (Waser *et al.* 1996; Ollerton *et al.* 2009). The structure of plant–pollinator communities is variable and depends on the composition of local pollinator fauna and on ecological interactions among coexisting plant species, which may in turn be subject to temporal change (Burkle and Alarcón 2011; Wolowski *et al.* 2017). The attempt to predict the main pollinators of a flowering plant in different ecological scenarios has raised questions regarding the validity of the ‘pollination syndrome’ concept (i.e. the entirety of floral traits associated with the attraction of, and effective pollination by, a functional group of pollinators; Fenster *et al.* 2004; also see Rosas-Guerrero *et al.* 2014; Ashworth *et al.* 2015), mainly because it could overlook other floral visitors and the role they play in the plant’s reproduction.

Sympatric flowering species must frequently share pollinators (see Mitchell *et al.* 2009). Pollinators can be a limited resource for the sexual reproduction of a plant, and such sharing could decrease individual reproductive success (Armbruster and Herzig 1984; Campbell 1985) through the effects of different forms of competition (Waser 1978). Evolution should favour adaptations that act to reduce heterospecific pollen deposition (Ashman and Arceo-Gómez 2013) as well as pollen misplacement during the visits to foreign stigmas (male fitness component; Morales and Traveset 2008; Muchhala *et al.* 2010). In this sense, sharing pollinators could promote a divergence in phenotypic characteristics related to attracting pollinators (Feinsinger 1987; Muchhala and Thomson 2012). However, pollinator sharing could also be advantageous to the plant community, since co-flowering plants could attract and maintain local populations of pollinators over the course of the year (facilitation; Schemske 1981; Moeller 2004; Ghazoul 2006; Sargent and Ackerly 2008).

The factors that influence plant–pollinator interactions may vary temporally and spatially, but the most common outcome of pollinator sharing among zoophilous plants is a reduction in conspecific pollen deposition (Morales and Traveset 2008). Several factors may reduce the costs of sharing pollinators among plants, including different microhabitat preferences (i.e. Kay 2006), staggered phenology (Feinsinger 1978; Lobo *et al.* 2003; Araujo *et al.* 2004;Aizen and Vázquez 2006; Kudo 2006), daily partitioning of floral rewards (i.e. Stone *et al.* 1998; Ramalho *et al.* 2014) and different pollinator foraging periods (Armbruster and Herzig 1984; Raine *et al.* 2007; Hoehn *et al.* 2008; Franco *et al.* 2011). Moreover, different floral morphologies in plants that share pollinators act to produce differential pollen placement on the body of the pollinator (Heinrich 1976; Armbruster *et al.* 1994; Kay 2006; Botes *et al.* 2008) while other plants may encourage the visit of different pollinator species, but from the same functional group (i.e. different species of bees), each of them probably with slightly different visitation behaviour, a characteristic that may account for their differential role in the overall pollination of the plant species (Missagia *et al.* 2014; Ramalho *et al.* 2014).

Bats are important pollinators of many tropical and subtropical plant families (Tschapka and Dressler 2002; Fleming *et al.* 2009) that possess flowers well adapted to pollination by these animals (von Helversen 1993; von Helversen and Winter 2003). Bats may be considered as highly effective pollinators, since they can transfer greater quantities of pollen than most other flower visitors, such as hummingbirds (Muchhala and Thomson 2010). However, since they often also ingest pollen and frequently visit several plant species during a foraging bout (i.e. Heithaus *et al.* 1975), their efficiency as pollinators may sometimes be limited (Fleming and Sosa 1994). Hence, sympatric chiropterophilous plants employ various strategies to reduce the cost of sharing the local bat pollinators. These include a staggered flowering phenology (Sazima *et al.* 1999; Lobo *et al.* 2003; Cummings *et al.* 2014), differing times of anthesis through the night (Howell 1977) and differences in floral morphology and subsequent pollen placement on the body of the bat (Tschapka *et al.* 2006; Muchhala and Potts 2007; Muchhala 2008; Muchhala and Thomson 2012; Stewart and Dudash 2015, 2017). Other strategies include a different nectar production schedule (Heithaus *et al.* 1975; Fleming *et al.* 1996) or dissimilar energetic qualities of the nectar (Tschapka 2004).

Bromeliaceae is an almost exclusively Neotropical plant family (Benzing 2000), comprising >3900 species (Gouda *et al.* 2018). The main pollinators in this family are hummingbirds (Krömer *et al.* 2006), but chiropterophily seems to have evolved multiple times within the family. Three genera (*Pseudalcantarea*, *Stigmatodon* and *Werauhia*) might even be completely bat-pollinated (Aguilar-Rodríguez *et al.* 2019). Members of the Glossophaginae subfamily (Phyllostomidae), especially the bat genus *Anoura*, are frequently reported as pollinators of these bromeliads (Aguilar-Rodríguez *et al.* 2019).

Many bromeliad species are sympatric and share pollinators (i.e. Sazima *et al.* 1996; Varassin and Sazima 2000; Wendt *et al.* 2008). However, pollinator sharing in chiropterophilous bromeliads has only been studied in two genera in Brazil (*Alcantarea* and *Vriesea*; Martinelli 1994; Sazima *et al.* 1995, 1999; Wendt *et al.* 2008). These studies found that some bat-pollinated bromeliad species share only one or two bat species as pollinators, but they did not investigate in detail the pollination ecology of the studied species, other than the probable role of staggered phenology.

The aim of this paper is to study the floral phenology, pollinators and floral rewards of four sympatric bromeliads, which belong to three different genera *Pitcairnia*, *Pseudalcantarea* and *Werauhia*. The characteristics of the putative pollination syndrome of these species are reminiscent to chiropterophily, suggesting that all of them might be bat-pollinated. We therefore predict that bats are the pollinators of these bromeliads, and that they will possess similar adaptations in order to attract the animals (absence of ethological isolation; Grant 1994; Baack *et al.* 2015). We expect that these species would possess similar strategies reported for other chiropterophilous plants to decrease pollinator competition, such as staggered flowering or mechanical isolation (i.e. deposition of pollen on different parts of the body of the bat as a result of different floral morphology). Moreover, if the bromeliads share a flowering period, nightly separation of the time of anthesis (Howell 1977) could promote partitioning of the resident bat pollinators.

## Methods

### Study site

This work was conducted from August 2014 to March 2016, in the state of Veracruz, Mexico, at two sites in the Ejido Adolfo Ruiz Cortines (18°32′03.55″N, 95°08′18.54″W, 1034 m a.s.l. and 18°32′40.04″N, 95°09′2.20″W, 1082 m a.s.l.), located in the Los Tuxtlas Biosphere Reserve on the south-eastern slopes of the San Martín Tuxtla Volcano (San Andrés Tuxtla municipality). In both sites, all the studied bromeliad species occur naturally and in proximity to each other. Average annual temperature is 18 °C and average annual rainfall is ~4000 mm (Soto 2004). The rainy and hot season occurs from June to October, while windy and colder weather is frequent from December to February. The original vegetation is a humid montane forest within a matrix of secondary vegetation, which is now surrounded by a matrix of secondary vegetation, as well as by agricultural cropland and cattle pastures (Castillo-Campos and Laborde 2004). One of the studied species (*Werauhia noctiflorens*) is very rare, and thus some individuals had to be translocated from other forest fragments near the study sites. These were placed on tree trunks at a height of ~1.50 m, which is similar to that of other individuals that have been found. Some individuals of *Pseudalcantarea viridiflora* and *Werauhia nutans* were relocated at a lower height in order to facilitate the study. At around 20 m from the first study site, we placed a ‘plant house’ with a mesh, in order to isolate some bromeliad individuals from pollinators while still exposing the plants to the same environmental conditions of light, temperature and humidity.

### Studied species

**Figure 1.**
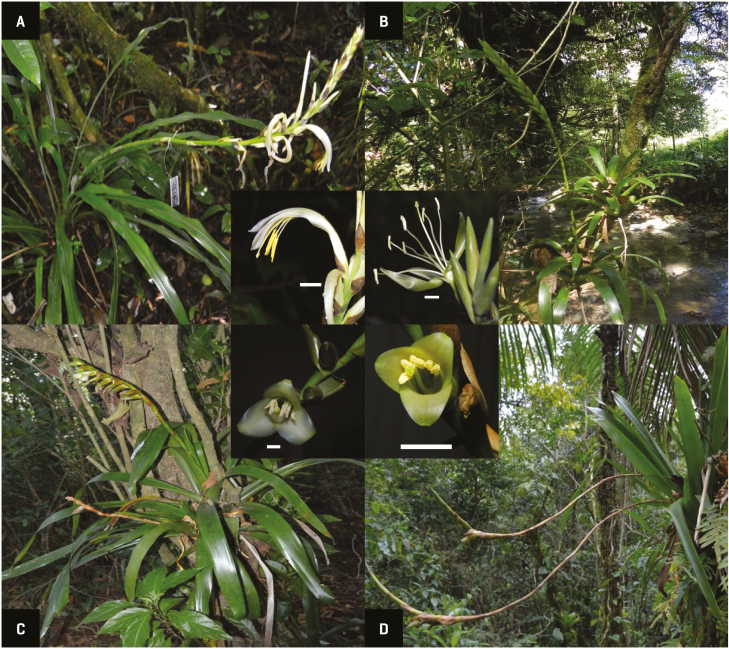
Study species in their habitat and with details of the flower. (A) *Pitcairnia recurvata*. (B) *Pseudalcantarea viridiflora*. (C) *Werauhia noctiflorens*. (D) *Werauhia nutans*. White bar corresponds to 1 cm. Photos: Pedro Adrián Aguilar-Rodríguez.

*Pitcairnia recurvata* is a terrestrial bromeliad from the Pitcairnioideae subfamily, which reaches a height of ~0.8–2 m (Espejo-Serna *et al.* 2005). It has zygomorphic flowers with a curved corolla and whitish petals. This species is widely distributed and abundant in the study area (Fig. 1A). *Pseudalcantarea viridiflora* is an epiphyte of ~0.65–1 m in height (Krömer *et al.* 2012), the leaves of which form a medium-sized tank for water storage. Its flowers are actinomorphic, presenting a helicoiform and subsessile corolla with greenish petals (Fig. 1B). *Werauhia noctiflorens* is an epiphytic bromeliad of up to 0.70–0.90 m that also has a medium sized tank (Krömer *et al.* 2007), but zygomorphic cup-like flowers with white to green petals (Fig. 1C). Finally, *W. nutans* is a small epiphytic bromeliad, which reaches ~0.30–0.60 m in height (Espejo-Serna *et al.* 2005) and also has a zygomorphic cup-like corolla with white to green petals (Espejo-Serna *et al.* 2005) (Fig. 1D). These four bromeliads show many floral characteristics generally found in chiropterophilous bromeliads (e.g. whitish to greenish corollas, faint odour, crepuscular anthesis and hexose-rich nectar; Krömer *et al.* 2007, 2008; Aguilar-Rodríguez *et al.* 2019).

### Phenology and anthesis

We monitored flowering in the four species through bimonthly visits to the study site during 2014 and 2015. The *in situ* experiments and pollinator assessment were conducted during the flowering peak of each species. For each species, we monitored at least 10 individuals from development of the floral buds to appearance of the fruit capsules. We marked all observed plants, as well as their buds, with a permanent marker on the floral bracts. We recorded changes in flower morphology, time of anthesis (i.e. at full opening of the corolla, with the gynoecium completely turgid, so that the flower can receive a visit from a pollinator), flower senescence and time of anther dehiscence (Martinelli 1994; Cascante-Marín *et al.* 2005). Furthermore, to estimate the duration of stigma receptivity, we recorded changes in the colour of the stigma and turgidity of the style by adding drops of hydrogen peroxide every 2 h after the beginning of the anthesis, until there was no perceivable bubble formation on the stigma surface (Martén-Rodríguez and Fenster 2008).

### Breeding system

To assess the breeding system of the four bromeliads, we performed four standard pollination treatments with at least 10 flowers per treatment per species. The individuals of the epiphytic species were planted in pots with gravel and placed in a ‘plant house’ in order to exclude all visitors. For *P. recurvata*, we covered the stigma in the field using small plastic tubes (drinking straw pieces) of ~2 cm in length **[see****Supporting Information—Fig. S1****]**, which were attached to the style with cotton thread at both ends (Aguilar-Rodríguez *et al.* 2014). The treatments were (i) spontaneous self-pollination (a non-manipulated flower), (ii) self-pollination, (iii) cross-pollination (see details in Aguilar-Rodríguez *et al.* 2014) and (iv) a control treatment (flowers exposed to pollinators) as a reference.

We quantified the number of fruits developed in order to calculate the fruiting success (fruit set) of each treatment, and then manually counted the number of seeds produced. The fruit capsules from *P. recurvata* contain many small seeds (~2000–3000), which makes manual counting impractical. In order to estimate the number of seeds for each treatment only in this species, we therefore used seed mass as a surrogate of seed set (Martén-Rodríguez and Fenster 2008) by using an analytical lab balance (Model: Pioneer PA124, readability: 0.1 mg, repeatability: 0.1 mg; Ohaus, USA) to weigh 1000 seeds in g (from 30 different Control fruits, from 10 individuals), and then use this value to estimate the number of seeds in the fruits of *P. recurvata*. We only weighted the seeds by themselves, without any adjacent tissue from the fruit capsule.

Since the preliminary exploration of the data suggested a non-normal distribution, a Kruskal–Wallis test with Tukey type comparisons (Dunn’s method) was used to assess differences between fruit and seed set from different treatments in all of the species (Paggi *et al.* 2007; Schmid *et al.* 2011a). Any treatment that produced no fruit as a result was excluded from the analysis. Due to stochastic events during the fieldwork, including poor climatic conditions as well as some human interference with the studied individuals, the final number of manipulated flowers was unbalanced among treatments and species. For all analyses, we used SigmaPlot ver. 12 and Statistica version 7.

To determine the self-compatibility of each species, we used the index of self-incompatibility (ISI; dividing the mean seed set of Autogamy by the mean seed set of Xenogamy). In this index, values between 0.30 and 1.00 denote self-compatibility (Zapata and Arroyo 1978; Wendt *et al.* 2001; Kamke *et al.* 2011). To determine if any of the bromeliads suffered from limited pollen supply, the pollen limitation index (PLI) was calculated as shown in Larson and Barret (2000) and Becker *et al.* (2011). Negative values denote pollen limitation, i.e. more seeds in the Control fruits than in the Xenogamy fruits.

### Nectar

The nectar production pattern during anthesis was determined for each bromeliad species. We used microcapillary tubes (10 and 80 µL) to extract the accumulated nectar from a flower every 2 h, beginning 2 h after anthesis and continuing until production decreased to zero. We also measured nectar sugar concentration using a hand-held refractometer (Model HRT32, range: 0–32 %, weight/weight, precision: 0.2 %; A. Krüss Optronic, Germany). To obtain the total nectar volume per flower, we summed the partial nectar volumes (Tschapka and von Helversen 2007) and also calculated the quantity of sugar contained in the nectar, using conversion tables (Kearns and Inouye 1993; Galetto and Bernardello 2005).

While this approach does not assess the non-sugar constituents of the nectar (Inouye *et al.* 1980), the quantity of sugar in the nectar is a close approximation of the energetic value of the reward offered by each species to their pollinators. To include plant density, we calculated the relative abundance of the four studied bromeliads by counting the flowering individuals in five randomly selected plots each of 20 m × 10 m (200 m^2^ per plot, modified from Krömer and Gradstein 2003) in natural forest fragments of the study area. Furthermore, we counted the flowering individuals in four transects of 2 m × 20 m (200 m^2^ per transect) along a trail that delimits the community of Ejido Adolfo Ruiz Cortines. We only counted epiphytic species growing below 5 m in height on the trees, and only those for which we could determine the species. The obtained density values were multiplied by the mean mg of diluted sugar produced by species (transformed to kJ with a conversion factor of 15.91 kJ; Winter *et al.* 1993), and then divided by the number of days among subsequent flowers in the same individual, calculated for each species (see data in the ‘Results’ section below), assuming that each bromeliad species produces one flower per night on average, in order to facilitate comparisons among the four species.

Due to the wet climatic conditions that prevailed during most of this study, the nectar measurements for *P. viridiflora*, *W. noctiflorens* and *W. nutans* were taken in a greenhouse in Xalapa, Veracruz, under similar conditions of humidity and light. All plants were moved into the plant house several weeks before the beginning of flowering in order to facilitate acclimation and reduce the stress for the plants. No further treatments were applied to these plants. We compared the nectar volume and mg of diluted sugar of the nectar between species, using a Kruskal–Wallis test (Fleming *et al.* 1996).

### Capturing and recording pollinators

To identify the potential pollinators of the bromeliads, we captured bats (authorized under permit number 01953/14 from the Secretaría del Medio Ambiente y Recursos Naturales) using two mist-nets (6 × 2.6 m and 12 × 2.6 m). The nets were set up close to flowering individuals (Martinelli 1994; Kaehler *et al.* 2005; Tschapka and von Helversen 2007) or along trails potentially used by bats as flight paths (Wilson *et al*. 1996), including the trail in which the number of flowering bromeliads was quantified.

At least one net was kept in close proximity (1–1.5 m) to the flowering bromeliads every night. We avoided netting during the full moon (Morrison 1978) and for more than three consecutive nights. After 2 h without capture, trapping was stopped (Santos-Moreno *et al.* 2010). Sampling effort was quantified as suggested by Straube and Bianconi (2002) in m^2^ h^−1^. In order to increase the sample size for potential bat visitor species, we also used a harp-trap in the entrance of a cave that serves as a roost for many bat species.

All captured bats were identified using the keys provided in Medellín *et al.* (2008) and Reid (2009) and subsequently released. We followed the taxonomical classification of Simmons (2005), Mantilla-Meluk and Baker (2010) and Cirranello *et al.* (2016). For all bats, we registered sex, age, weight (g) and forearm length (mm).

We searched for pollen carried in the fur of the bats, especially on the head, back or breast areas, as well as on the wings (Caballero-Martínez *et al.* 2009). We then collected the visible pollen with a moist brush and rinsed the bristles in 2 mL Eppendorf tubes with at least 1.5 mL of 70 % ethanol (Voigt *et al.* 2009). We took a reference sample from the anthers of each bromeliad species. We used a microscope at ×100 and ×400 (Zeiss, Germany, Model: 426126) for comparing pollen with the samples taken from the bodies of the bats (Herrera and Martínez-del-Río 1998; Muchhala and Jarrín-V 2002). We analysed the samples using presence/absence categories (Voigt *et al.* 2009). For this, we agitated each vial in order to resuspend the grains, extracted six drops and analysed these under the microscope. For each drop, we counted the pollen grains using the ‘scan’ technique (Caballero-Martínez *et al.* 2009) and thus determined the frequency of bromeliad pollen. The bromeliad pollen could be easily distinguished from the pollen of other families present in the samples, but it is difficult with some genera at subfamily level (Silva *et al.* 2016; Souza *et al.* 2017). In this case, the only overlapping species from the same genus were both species of *Werauhia*, and no bat was captured at that moment. Each sample was categorized as positive for bromeliad pollen when we found at least three bromeliad pollen grains in the examined drops (Heithaus *et al.* 1975). Finally, we summed all of the positive samples and calculated the percentage of bromeliad pollen presence.

We directly observed flowers (of each species) for at least 12 h during daylight (early morning to late afternoon), to check for diurnal floral visitors as possible pollinators. In addition, we recorded the nocturnal floral visitors of the four bromeliads in the field, using an infrared-sensitive camera (DCR-SR65; Sony Corporation, Japan) with an infrared light (HVL-HILR; Sony Corporation, Japan), as well as a modified Go Pro Hero 3 cam (Go Pro Inc., USA) with attached infrared LED lights. The cameras were placed on a tripod at a height of 1.3–1.5 m from the ground, and ~1–1.5 m from the open flower. Due to the unfavourable weather conditions during most of the fieldwork, as well as technical constraints, the cameras only recorded for 3–3.5 h per night. However, this period encompassed at least the first peak of nectarivorous bat activity during the night (Heithaus *et al.* 1974, 1975; Quesada *et al.* 2003), 3 h after sunset (typically from 1900 to 2200 h).

We considered a floral visitor, in its broadest meaning, to be any animal that could make contact with the floral parts (*sensu*Schmid *et al.* 2011b); however, in order to be considered a legitimate pollinator, an animal had to make physical contact with the reproductive parts of the flower. By introducing the head into the corolla, bats tend to make contact with the anthers and stigma (see Slauson 2000). For each group of animals, we determined the frequency of visits (in visits per flower per hour; Schmid *et al.* 2011a), the number of legitimate visits and the behaviour during each visit (approach, number of flowers visited and the sequence on the inflorescence, taking of rewards other than nectar; Montalvo and Ackerman 1986; Martinelli 1994). The duration of 10 randomly selected hovering visits to each bromeliad species was evaluated using the program Adobe Premiere CS5 (Adobe Systems Incorporated, USA), by reducing the original speed of the recordings to 10–13 % of normal speed. We determined the duration by quantifying the number of frames where the mouthparts were in the corolla. We inferred the bat species recorded in the videos based on the relative size of the bats in relation to the flower, in addition to visible morphological characteristics, such as the presence of an uropatagium, and their behaviour during the visit (to differentiate frugivorous, non-hovering species from nectarivorous species that approach flowers by hovering flight; Fischer 1994).

## Results

### Phenology and anthesis

**Figure 2.**
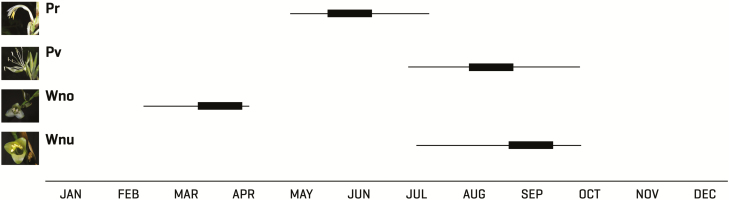
The flowering periods of the studied bromeliads: Pr: *Pitcairnia recurvata*, Pv: *Pseudalcantarea viridiflora*, Wno: *Werauhia noctiflorens* and Wnu: *Werauhia nutans*. Bold lines indicate the peak of the flowering period.

The flowering peaks of the studied species presented little overlap, although it is possible to find individuals of three species flowering during the rainy season (June to October) (Fig. 2). The first species to flower was *W. noctiflorens*, beginning at the end of February and extending into March–April. Some individuals of *W. nutans* began to flower between March and April, particularly at higher elevations (1220 m a.s.l.), but the main flowering period for this species began in June and lasted until October. *Pitcairnia recurvata* initiated flowering in mid-May and flowered until mid-July. Flowering in *P. viridiflora* began at the end of July and lasted until October, but most individuals flowered during September. Over the 2 years of observation, the flowering period remained largely constant for most species, with the exception of *W. nutans*, for which the period varied over several months between years.

*Pitcairnia recurvata* individuals presents 26 ± 9.05 flowers (coefficient of variation (CV): 34.08 %; range: 14–46 flowers, *n* = 34 individuals) over its flowering time, producing one (maximum three) flower every 1.53 ± 0.73 days. Time of anthesis in this species is around 2100 h, which is one and a half hours after sunset at the time of flowering. The upper petal protrudes above the two others, and the stamina hang down, with the style, situated in parallel to the lower petals. The stigma seems to already be receptive by the time the petals open completely. No conspicuous floral scent was perceived. A newly opened flower prevents self-pollination by maintaining a distinct separation between the stigma and the anthers. The style withers at around 0600 h, hanging even lower than the stamina when newly opened. At this time, 9–10 h after anthesis, the stigma points downwards and this spatial arrangement again prevents any contact with the anthers.

The inflorescence of *P. viridiflora* presents 15.40 ± 3.44 flowers, (CV: 22.34 %; range: 10–22 flowers, *n* = 10 individuals), producing one (maximum two) flower every 2.09 ± 1.20 days. The flower opens at around 1900 h, ~20 min before sunset. Pollen is already available at the time of the anthesis, and some grains are already deposited on the surface of the still unreceptive stigma. Stigma receptivity begins 20–30 min later when the stigma gets visibly wet. The floral parts lose turgidity (especially the style), and the stigma becomes dark in color at 15–17 h after anthesis.

An inflorescence of *W. noctiflorens* presents a total of 6.73 ± 1.67 flowers (CV: 24.81 %; range: 3–9 flowers, *n* = 15 individuals), producing one (maximum two) flower every 1.21 ± 0.42 days. The time of anthesis is around 1800 h, nearly 30 min before sunset during peak flowering. Dehiscence of anthers occurs rapidly following anthesis, while the stigma is receptive between 20 and 30 min after anthesis, as shown by the wet appearance of the stigma. A ‘sweat-like’ odour is slightly noticeable with the beginning of anthesis, but it only is highly perceptible at ~2 h after anthesis. The corolla closes at around 0600 h, nearly 12 h after anthesis. At this time, with the petals already closed, the stigma touches the anthers.

*Werauhia nutans* presents 8.02 ± 2.62 flowers per inflorescence (CV: 32.66 %; range: 3–14 flowers, *n* = 45 individuals), producing one flower every 3.87 ± 1.21 days, which opens at around 1940 h, nearly 10–15 min after sunset. As with the flowers of *W. noctiflorens*, dehiscence of anthers occurs right after the anthesis, followed by stigma receptivity <30 min after. Only a faint odour is perceptible throughout the night. The flowers close 10–12 h later, contacting the stigma as in *W. noctiflorens*.

### Breeding system

We found that all four bromeliad species are self-compatible, producing seeds regardless of the origin of the experimentally provided pollen (self- or cross-pollination; **see****Supporting Information—Table S1**) (ISI > 0.30). The results indicate that, in any case, there was no difference in seed sets among treatments, including the natural seed set of a flower exposed to natural pollinators (*P. recurvata*: *H* = 1.275, df = 2, *P* = 0.529; *P. viridiflora*: *H* = 5.904, df = 3, *P* = 0.116; *W. noctiflorens*: *H* = 5.223, df = 3, *P* = 0.156; *W. nutans*: *H* = 0.959, df = 3, *P* = 0.811). However, the natural fruit set of all species does not reach 80 %, regardless of their self-compatibility. *Pitcairnia recurvata* seems to depend on the visit of a pollinator to produce seeds and develop fruits. Interestingly, the PLI indicates that *P. recurvata* is not pollen limited, as with *W. nutans*, but *P. viridiflora* and *W. noctiflorens* seem to be limited (positive values in PLI). Table 1 summarizes the results of the pollination experiments designed to determine the breeding systems of the four bromeliads.

**Table 1.** Results from the pollination treatments conducted to determine the breeding system of the four studied bromeliads. Values in ISI above indicate self-compatibility. Positive numbers in PLI indicate pollen limitation in the species (i.e. the species does not reach it maximum seed set in its natural environment).

Treatments	Species (ISI/PLI)	No. of flowers	Fruit set (%)	Seed set (mean ± SD)
Spontaneous self-pollination	*Pitcairnia recurvata*	15	0	_
	*Pseudalcantarea viridiflora*	14	64.29	1165.67 ± 358.47
	*Werauhia noctiflorens*	19	94.74	1430.89 ± 659.38
	*Werauhia nutans*	21	80.95	364.66 ± 331.55
Cross-pollination	*Pitcairnia recurvata*	14	57.14	1881.88 ± 891
	*Pseudalcantarea viridiflora*	16	75	967.58 ± 354.10
	*Werauhia noctiflorens*	19	68.42	1288.54 ± 397.62
	*Werauhia nutans*	14	57.14	426.75 ± 367.98
Self-pollination	*Pitcairnia recurvata*	14	50	2459.00 ± 920.14
	*Pseudalcantarea viridiflora*	14	78.57	930.36 ± 485.96
	*Werauhia noctiflorens*	14	71.43	1291.00 ± 849.77
	*Werauhia nutans*	17	64.71	406.64 ± 307.98
Control	*Pitcairnia recurvata* (1.31/−0.13)	20	75	2130.13 ± 778.83
	*Pseudalcantarea viridiflora* (0.96/0.52)	19	68.42	468.46 ± 340.87
	*Werauhia noctiflorens* (1.00/0.12)	25	72	1125.67 ± 571.60
	*Werauhia nutans* (0.95/−0.60)	20	50	680.90 ± 349.02

### Nectar

**Figure 3.**
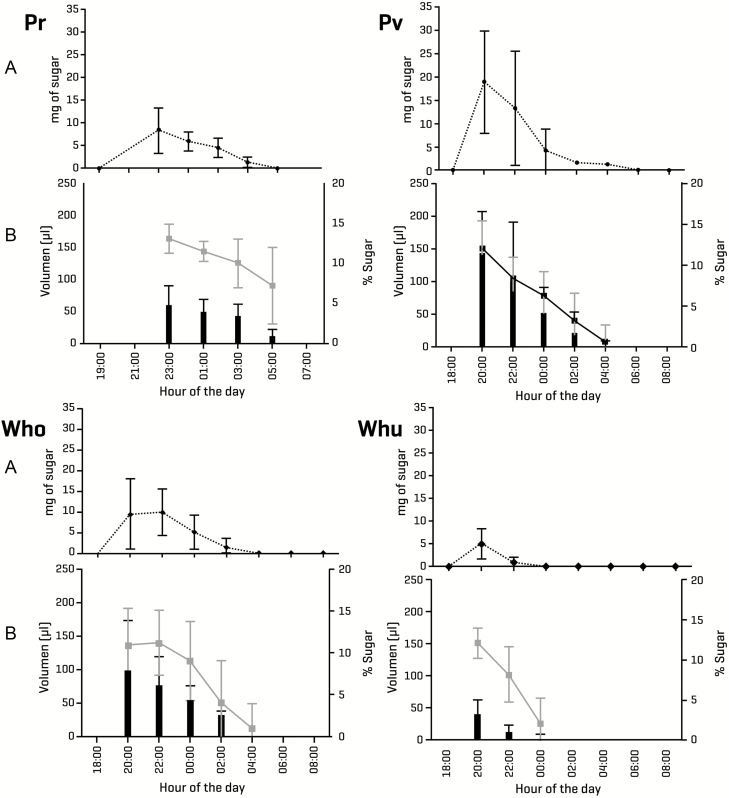
Mean nectar values (A) mg of diluted sugar: dotted black line; (B) volume: black bars, sugar concentration: gray line; SD: vertical lines of the four bromeliad species studied: Pr: *Pitcairnia recurvata*, Pv: *Pseudalcantarea viridiflora*, Wno: *Werauhia noctiflorens*, Wnu: *Werauhia nutans*.

Nectar production varies highly among the four studied bromeliad species. The overall pattern shows that the highest nectar volume and concentration both occur in the early hours of the evening, following anthesis. Even in *P. viridiflora* and in both *Werauhia* species, which all open their flowers during the afternoon, nectar production is strictly nocturnal (Fig. 3).

Although there are significant differences in the nectar traits among the four species (Table 2; **see****Supporting Information—Table S1**), the overall values are broadly similar among most species. *Pseudalcantarea viridiflora* and *W. noctiflorens* produce nectar in similar quantity and of comparable quality, while *P. recurvata* shows values that fall in between those of the former two species. In contrast, *W. nutans* plants produce nectar in the lowest quantity and of the lowest quality (less volume, extremely dilute and therefore with a very low sugar content).

**Table 2.** Mean nectar traits of the four bromeliads studied. At the bottom, the Kruskal–Wallis test results; different letters in superscript indicate differences found in *post hoc* test at *P* < 0.05.

Species	Nectar volume per flower (μL; mean ± SD)	Concentration (%; mean ± SD; CV)	Sugar production per flower (mg, mean ± SD; CV)	Energy density in the habitat (kJ ha^−1^ day^−1^)
*Pitcairnia recurvata* (*n =* 10 flowers, 7 individuals)	165.56 ± 50.29^a^; CV: 30.38 %	8.37 ± 1.29^a^; CV: 15.41 %	20.18 ± 6.81^a^; CV: 33.75 %	212.76
*Pseudalcantarea viridiflora* (*n =* 11 flowers, 4 ind)	328.02 ± 179.96^a^; CV: 54.86 %	6.12 ± 2.78^bc^; CV: 45.42 %	38.34 ± 26.11^a^; CV: 68.10 %	16.61
*Werauhia noctiflorens* (*n =* 10 flowers, 4 individuals)	303.65 ± 153.58^a^; CV: 50.58 %	11.64 ± 13.17^ac^; CV: 113.14 %	35.29 ± 14.82^a^; CV: 41.99 %	23.19
*Werauhia nutans* (*n =* 10 flowers, 7 individuals)	56.89 ± 26.10^b^; CV: 45.88 %	3.72 ± 1.08^b^; CV: 29.03 %	6.78 ± 3.77^b^; CV: 55.60 %	74.32
	*H* = 26.411, df = 3, *P* < 0.05	*H* = 23.916, df = 3, *P* < 0.05	*H* = 23.803, df = 3, *P* < 0.05	

The density of the four bromeliad species in their natural habitat differed widely. *Pitcairnia recurvata* has a density of 1011 individuals per ha, while *P. viridiflora* has 106 individuals per ha. The most abundant species is *W. nutans*, with 1439 individuals per ha, and the least abundant is *W. noctiflorens*, with 50 individuals per ha. Considering these data, the estimated values for energy density in the habitat are as follows: *P. recurvata* offers ~212.76 kJ ha^−1^ day^−1^; *P. viridiflora*: 16.60 kJ ha^−1^ day^−1^; *W. noctiflorens*: 23.19 kJ ha^−1^ day^−1^; and *W. nutans*: 74.32 kJ ha^−1^ day^−1^.

### Capture and recording of bats

We captured 144 bat individuals, distributed in 19 species belonging to three families, with a sampling effort of 53 757 m^2^ h^−1^ over 33 nights, and one night of harp-trapping over 4 h. These nights were distributed over the peak of the flowering season for each species (August–September for *P. viridiflora* and *W. nutans*, June for *P. recurvata*, and March for *W. noctiflorens*). The bat species captured were: Momoopidae: *Mormoops megalophylla* (1 individual), *Pteronotus parnellii* (1); Phyllostomidae: *Anoura geoffroyi lasiopyga* (7), *Artibeus jamaicensis* (6), *Artibeus aztecus* (1), *Artibeus toltecus* (59), *Carollia sowelli* (11), *Desmodus rotundus* (20), *Diphylla ecaudata* (2), *Glossophaga soricina* (8), *Hylonycteris underwoodi* (1), *Sturnira hondurensis* (10); Vespertilionidae: *Bauerus dubiaquercus* (2), *Eptesicus furinalis* (1), *Myotis* cf. *auriculus* (1), *Myotis* cf. *californicus* (4), *Myotis elegans* (1), *Myotis keaysi* (2) and *Myotis nigricans* (6).

**Figure 4.**
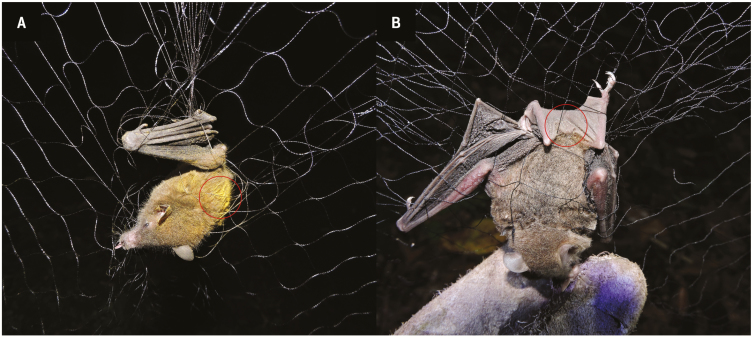
Nectarivorous bats carrying pollen captured during this study. (A) *Anoura geoffroyi* captured near blooming individuals of *Pitcairnia recurvata*; (B) *Glossophaga soricina* captured in proximity to flowering *Pseudalcantarea viridiflora*. Circles highlight the location of pollen on the bats’ body. Photos: Pedro Adrián Aguilar-Rodríguez and M. Cristina MacSwiney G.

We found pollen on the fur and/or wings of *A. geoffroyi*, *G. soricina* and *H. underwoodi*. These three species belong to the specialized nectarivorous Glossophaginae subfamily of the Phyllostomidae and were captured during the flowering of *P. recurvata* and *P. viridiflora*. No nectarivorous bat was captured during the flowering of *W. noctiflorens*, although visits to the plant were recorded with the camera (see Table 3). The pollen from both species was easily distinguishable between them by using the reference pollen collected directly from flowering plants. In addition, at the site, the flowering of both bromeliads did not overlap. *Anoura geoffroyi* individuals were captured during the flowering of both *P. recurvata* and *P. viridiflora*. At *P. recurvata*, three out of five of the captured individuals carried abundant pollen on the dorsal region (Fig. 4A). During the flowering of *P. viridiflora*, the pollen loads were less conspicuous and found on the back and wings of two bats. *Glossophaga soricina* was only captured during the flowering of *P. viridiflora*, and pollen was mainly carried on its uropatagium (Fig. 4B). *Hylonycteris underwoodi* was caught only on one night during the flowering of *P. viridiflora*, with a low quantity of pollen found on its uropatagium. The three *A. geoffroyi* individuals captured during the flowering of *P. recurvata* carried 2883 ± 0.002 pollen grains, all from *P. recurvata*. In addition, one *A. geoffroyi* carried pollen from *P. viridiflora* (15 grains, 75 % of the total grains identified). Of the eight individuals of *G. soricina* that carried pollen, only 30.77 % (12.63 ± 17.03 grains) came from *P. viridiflora*, meaning 70 % of the pollen grains were not from a member of the Bromeliaceae family (being *P. viridiflora* and *W. nutans* the only nocturnal bromeliads flowering at the site, with pollen grains easily discernible between them). In the samples of *H. underwoodi*, only three pollen grains could be identified, two of which corresponded to *P. viridiflora*.

**Table 3.** Bat species registered visiting the studied bromeliads. *Legitimate visits refer to the visits that contact reproductive parts of the flower. **The visits were selected randomly among the hovering visits by the bats.

Species	Pollinator	Legitimate visits/ visits* (%)	Visit duration (mean ± SD)**	Visitation frequency (visits per flower per hour)
*Pitcairnia recurvata*	*Anoura geoffroyi*	30/35 (85.71)	0.38 ± 0.17 seg (*n* = 10 visits)	0.041
*Pseudalcantarea viridiflora*	*Glossophaga soricina/ Hylonycteris underwoodi*	100/146 (68.49)	1.12 ± 0.33 seg (*n* = 10 visits)	0.351
*Werauhia noctiflorens*	*Anoura geoffroyi*	22/23 (95.65)	0.51 ± 0.11 seg (*n* = 10 visits)	0.116
*Werauhia nutans*	None	–	–	–

A total of 131 h of video recordings were made in order to identify the pollinators of the four bromeliads. Based on the captured bats and the morphological characteristics of the recorded individuals, we suggest the possible pollinator species of the three bromeliads that received bat visits (Table 3; **see****Supporting Information—Video S1**–**S3**).

**Figure 5.**
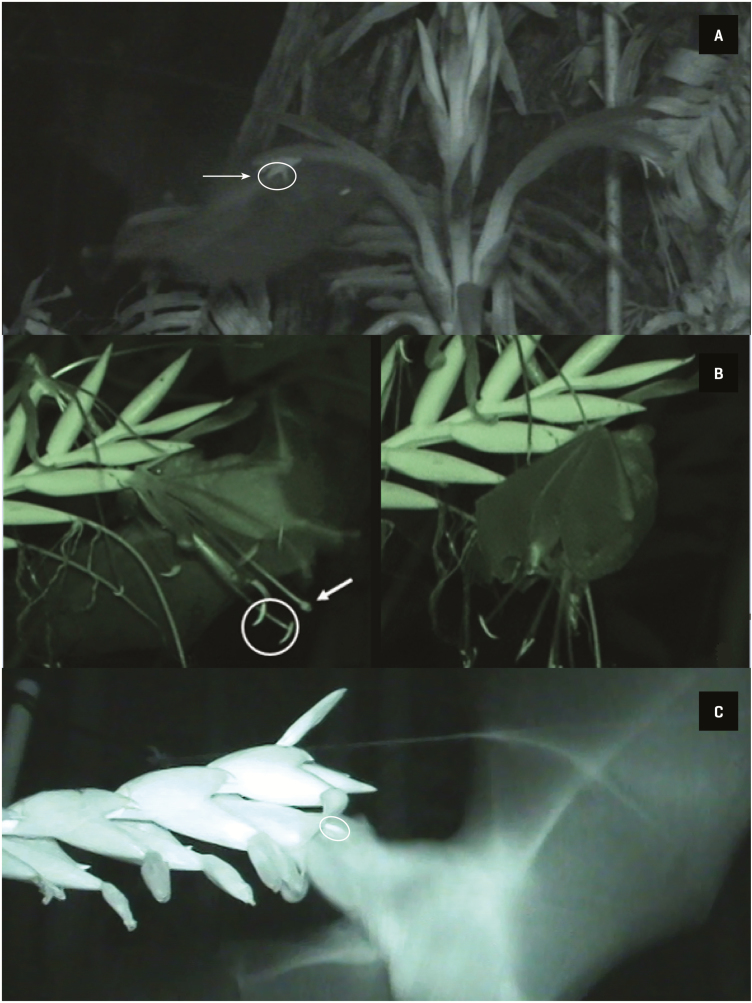
Bats pollinating the flowers of the studied bromeliads. (A) A bat, probably *Anoura geoffroyi*, visiting *Pitcairnia recurvata*. The circle denotes the anthers, and the arrow points towards the stigma. (B) A bat, probably either *Glossophaga soricina* or *Hylonycteris underwoodi*, visiting a flower of *Pseudalcantarea viridiflora*. Flower visit by hovering flight (left), the circle indicates some anthers, and the arrow points to the stigma. After arriving by hovering, *G. soricina* sometimes hangs from the flower (right), licking the nectar for about a second, before leaving. (C) A bat, probably *A. geoffroyi*, visiting *Werauhia noctiflorens*. The circle denotes the anthers.

*Anoura geoffroyi* was the only pollinator registered for *P. recurvata* in 41 h of recordings. During a visit, the bat first touches the distal part of the flower (the tip of the corolla), initially making contact with the stigma. Later, by following the corolla tube to reach the nectar at the base of the flower, it makes contact with the anthers with the upper portion of the head and in the nototribical portion of the body (Fig. 5A). When leaving, the bat drops from the flower, moving the whole inflorescence as a consequence. The whole visit takes less than a second.

We recorded the smaller bat species, either *G. soricina* or *H. underwoodi*, visiting *P. viridiflora* over 32 h of recordings, with the former of the two bat species the most likely visitor on most occasions according to our captures. These smaller bats approach the flower by hovering and push the whole inflorescence during its visit, which lasts around 1 s (Table 3). As a result of this movement, the uropatagium touches both the stigma and the anthers (Fig. 5A). Occasionally, the bats also hang down from the flowers after arriving; extracting the nectar and licking the pollen adhered to the wings before leaving (Fig. 5B).

We recorded probably *A. geoffroyi* visiting *W. noctiflorens* during this flowering period, in 28 h of recordings. During these visits, the bat approaches the flower and inserts its whole head into the corolla. Its forehead and cheeks thus make contact with the stigma and anthers (Fig. 5C). After about half a second, it pushes back its body and leaves the flower. The probable site where the pollen would adhere to this bat would therefore be on the forehead. The bat *A. geoffroyi* performed legitimate visits to both *P. recurvata* and *W. noctiflorens* (Table 3) in almost every approach, probably pollinating the flowers, whereas the smaller bats that visited *P. viridiflora* sometimes failed to pollinate it.

No bats were recorded visiting *W. nutans* in over 30 h of recordings. At least half of the recording effort for this species was made after the flowering period of *P. viridiflora*. Recordings covering flowers of *W. nutans* and *P. viridiflora* with the same camera failed to show any legitimate pollinator of *W. nutans*.

Many Halictidae bees have been recorded visiting the anthers of *P. recurvata* during the morning following anthesis; however, most of the stigmas had pollen at that time and the bees made only sporadic contact with the stigmas, some even trying to collect pollen from them. Considering that both *Werauhia* species close their corolla at sunrise, and that senescence changes the orientation of the stigma in both *P. recurvata* and *P. viridiflora*, in addition to our observations regarding the duration of stigma receptivity, diurnal floral visitors can be ruled out as pollinators of any of the studied bromeliads.

## Discussion

The four studied bromeliads occur sympatrically and share nocturnal flowering habits, with three of them confirmed as pollinated by bats, and one seemed to rely in selfing more than in the visit of pollinators as a reproductive system. Even considering that all species are self-compatible (ISI = ~0.90; Ferrer *et al.* 2009), they seem to have evolved different strategies to decrease competition for their bat pollinators, including a staggered flowering season, different pollen placement sites on the bodies of the bats and differences in the nectar offered as a reward. It is interesting, considering that at least the most frequent pollinators of *P. viridiflora*, small-sized nectarivorous bats, may be absent as a frequent visitor for either *W. noctiflorens* and the pollinator-dependent *P. recurvata*. Competition for pollinators occurs when at least one species suffers decreased reproduction because of sharing pollinators (Waser 1978), and the mechanisms that lead to this include a preference of pollinators for one plant species over the other, or interspecific pollen transfer (Morales and Traveset 2008). Sympatric bromeliads may segregate its flowering through the year to avoid competition, but floral morphology coupled with nectar offering might play an important role as well.

These differences could reflect the dependence of each bromeliad species on its pollinators. In an assemblage of sympatric bromeliads, pollen deposition could be important to the segregation of species (Wendt *et al.* 2001, 2008; Palma-Silva *et al.* 2015). Post-pollination barriers in Bromeliaceae are less effective among closely related species or even between species with ‘wet stigmas’ (Matallana *et al.* 2016), making the strategies to avoid competition and pollen misplacement to foreign stigmas very important to the overall fitness of the species. Self-compatibility is ancestral in Bromeliaceae (Wolowski and Freitas 2015) and an evolutionary reversal from self-crossing is unlikely in most angiosperms (Igic *et al.* 2008). In this sense, it can be expected that all of the studied species are self-compatible (but see *Encholirium* spp. in Christianini *et al.* 2013 and Hmeljevski *et al.* 2017).

While it is self-compatible, *P. recurvata* seems to be a facultative xenogamous species, as it seems to lack a mechanism for self-pollination (also see Table 1). This species presents a distinctive spatial separation between anthers and the stigma. This herkogamy prevents nocturnal self-pollination (P. A. Aguilar-Rodríguez, pers. obs.). By the time the floral parts lose their turgor, the stigma is already covered by pollen as a result of pollinator visitation. However, even in the morning, the stamina loses turgor later than the style, conserving the herkogamy even longer through the morning.

The results indicate that *P. recurvata* experienced no pollen limitation, and pollination in this species may thus be non-restricted at our study site, even if the species seems to be pollinator dependent. Furthermore, this species might occasionally experience self-pollination via geitonogamy since, on some nights, individual plants produced two or even three flowers simultaneously. We consider that *P. recurvata* is adapted to bat pollination (see also *Pitcairnia albiflos*; Wendt *et al.* 2001), taking into account the time of anthesis that excludes diurnal nectar thieves (see Christianini *et al.* 2013; Marques *et al.* 2015; Aguilar-Rodríguez *et al.* 2016; Queiroz *et al.* 2016) and the energetically high reward that may promote reliable bat visitation. The presence of diurnal floral visitors that are detrimental to the fitness of the plant, e.g. by wasting pollen (Thomson and Wilson 2008), might have reinforced the strong adaptation of *P. recurvata* towards chiropterophily. In addition, the reduction in nectar volume over the course of the night, also found in other chiropterophilous species from other families (Fleming *et al.* 2009), prompts a higher number of flower visits in order to meet the energetic needs of the bats (Howell 1979; Tschapka and von Helversen 2007).

Both *Pseudalcantarea* and *Werauhia* are genera that could be exclusively bat-pollinated (see Tschapka and von Helversen 2007 and Aguilar-Rodríguez *et al.* 2014 for other examples in these genera), but the species studied so far (including the ones in this study) are facultative autogamous, capable of self-pollination, which might serve as a strategy to guarantee reproduction (Stebbins 1957; Kennedy and Elle 2008; Busch and Delph 2012). Self-crossing may contribute to reproductive isolation by reducing heterospecific pollen transfer/deposition among species (Fishman and Wyatt 1999; Wendt *et al.* 2002; Matallana *et al.* 2010), since the deposition of pollen has important implications for overall fitness (Matallana *et al.* 2010; Ashman and Arceo-Gómez 2013).

Nevertheless, there are differences in terms of the time at which self-pollination occurs during anthesis. Both *Werauhia* species self-pollinate at the end of the anthesis when the corolla closes early in the morning. In the event of any cross-pollen grains reaching the stigma during the night, the pollen tubes will already have grown and reached the ovules (Martinelli 1994; Matallana *et al.* 2016). In contrast, *P. viridiflora* flowers usually already show their own pollen at the proximal portion of the stigma prior to anthesis, so it is less probable that they receive any other conspecific pollen before self-pollination occurs. This results in at least some ovules, that otherwise would have been available for cross-pollination, being self-pollinated (Lloyd 1992). This is important because *Werauhia* species and *P. viridiflora* fruit production via self-pollination occur at different moments of the floral lifespan, although this has a similar effect on the seed set since *W. noctiflorens* and *P. viridiflora* are both pollen limited at the study site. This limitation might be due to the lack of sufficient conspecifics in the vicinity to produce maximum seed set (through some form of self-depression; Lloyd 1992). However, many factors can cause pollen limitation, e.g. pathogens, nectar thieves, herbivory and seed predation (Gómez *et al.* 2010), being nectar thieves and herbivores quite common in *P. viridiflora*. In addition, quantifying pollen limitation at seed set may underestimate the strength of this phenomenon, since the cross-pollination treatments with supplemented pollen of various individuals could cause the plant to assign resources to the seeds of best quality (Gómez *et al.* 2010; Harder and Aizen 2010). For instance, *Werauhia gladioliflora* seems not to be pollen limited and is very abundant in a habitat with numerous bat pollinators (Cascante-Marín *et al.* 2005; Tschapka and von Helversen 2007).

A staggered flowering pattern could represent a diffuse facilitation between co-flowering plants that share a limited number of pollinators, since a constant supply of food can help to maintain a stable pollinator community (Feinsinger 1987; Cortés-Flores *et al.* 2017) and bat-pollinated species present longer flowering periods than species with other pollinators (see Cortés-Flores *et al.* 2017). Allochronic isolation among co-flowering plants is likely to occur in communities under strong pollen limitation (Devaux and Lande 2009) and two of the studied species (*W. noctiflorens* and *P. viridiflora*) are separated by several months in their flowering peaks.

Different floral designs in chiropterophilous plants produce differing patterns of pollen deposition on their bat pollinators (e.g. Stewart and Dudash 2016), which is crucial for correct pollen transfer (Stewart and Dudash 2017). Since the floral morphology of *P. viridiflora* is very different from that of *Werauhia*, there is a mechanical isolation between these genera: *Werauhia* deposits pollen on and receives pollen from the forehead of the bats, whereas *P. viridiflora* utilizes the uropatagium (see Fig. 5) or the wings (as evidenced by the captured individual of *A. geoffroyi*).

*Werauhia nutans*, a very abundant species with no pollen limitation, may be synchronopatric with other chiropterophilous species by using self-pollination and clonal reproduction as its principal reproductive strategies. Only *W. nutans* has a major overlap with all other species, since some individuals flower early in the year, being synchronopatric with the last flowering individuals of *W. noctiflorens*. Later in summer, *W. nutans* flowering overlaps strongly with the end of the flowering season of *P. viridiflora*. However, no nectarivorous bats were captured nearby any flowering plant of *W. nutans*, neither recorded, even if similar sampling effort were used for each species. Since no bat visits were recorded in *W. nutans*, we have no direct evidence for competition between these species, although it is likely to exist. Based on floral morphology, pollen deposition on *W. nutans* should correspond to the pattern in *W. noctiflorens*, and thus there is no guarantee of mechanical isolation among these closely related species. Competition mediated by pollinators may potentially also influence self-pollination in a species sharing pollinators with other species (Mitchell *et al.* 2009 and references therein), favouring the self-compatible breeding system (Fishman and Wyatt 1999). This is consistent with its relatively small floral parts in relation to other members of the genus, which may favour self-pollination (Jarne and Charlesworth 1993), as well as the poor nectar reward offered by individual plants. Both *W. noctiflorens* and *P. viridiflora* have a higher nectar production than *W. nutans*, and bats might therefore favour the former species, even where *W. nutans* is highly abundant. The nectar volume of <60 μL per night in *W. nutans* is extremely low for bat-pollinated bromeliads and might not entirely cover the energetic cost for a visiting bat. We suggest that *W. nutans* largely favours self- over cross-pollination, and saves resources by producing less nectar than the other, more bat-dependent species. Perhaps the occasional visit by bats, which we did not observe in this study, is sufficient to maintain the genetic variability within the population.

One of the main questions resulting from our results is about why, at first glance, the small nectarivorous bats are absent from the recordings of both *P. recurvata* and *W. noctiflorens*, but frequently recorded as visitors of *P. viridiflora*. Both *A. geoffroyi* and *G. soricina* visit many flowering species (Sánchez-Casas and Álvarez 2000; Caballero-Martínez *et al.* 2009), but the ‘core nectarivorous bats’ at higher elevations are comprised by the genus *Anoura* (Fleming *et al.* 2009). We know, at least, that *G. soricina* and *H. underwoodi* are present during September–October because of our mist-netting trapping results, and that *A. geoffroyi* is present almost year around. Comparing the video recordings from *P. viridiflora* from Los Tuxtlas with recordings of the same species being visited by *A. geoffroyi* in another location in central Veracruz, Mexico (P. A. Aguilar-Rodríguez *et al.*, unpubl. data), we discarded that the bats recorded visiting *P. viridiflora* during this study were *Anoura*, and likely, either *G. soricina* or the less abundant *H. underwoodi*.

Mechanical isolation and time of anthesis might play roles in partitioning of the nectarivorous bat species between *P. recurvata* and *P. viridiflora* in the studied area. The nectar extraction efficiency of *G. soricina* is reduced when this species visits flowers with a nectar tube (González-Terrazas *et al.* 2012; see also Tschapka *et al.* 2015), and the corolla morphology of *P. recurvata* contrasts with the open corolla of *P. viridiflora* that exposes the nectar to any floral visitor (Fig. 1A and B). The floral morphology of *P. recurvata* alone could at least limit this bat from utilizing this bromeliad. Indeed, when the nectar production of *P. viridiflora* decreased later at night, *G. soricina* exhibited a perching behaviour on the inflorescence (Fig. 5B), which might reflect the increased efforts of this species to access the nectar deep down in the calyx. On the other hand, environmental conditions have an effect on the presence and activity patterns of pollinators (Sánchez-LaFuente *et al.* 2005). The larger *Anoura* species are particularly well adapted to the nocturnal climatic conditions at higher elevations (Soriano *et al.* 2002). This also may allow *A. geoffroyi* to visit *P. recurvata* flowers that open late at night, in contrast to other bromeliads that are crepuscular (see Aguilar-Rodríguez *et al.* 2016, 2019), like *P. viridiflora*. Other explanation could be the specific nectar requirements for each bat species in relation to the nectar offered by the different bromeliads and the competition with other bats for it. It is notable that, taken together the density of plants and the amount of nectar produced, *P. recurvata* offers ~10 times more energy in its nectar than *W. noctiflorens* and *P. viridiflora*, even though these present a high sugar production per flower (Table 2). Being more abundant and growing in clumps, *P. recurvata* might be a more reliable and less competed nectar source for a bigger nectarivorous bats (and hence, with bigger energetic demands) than *P. viridiflora*.

A total of seven chiropterophilous bromeliads occur in the humid montane forest of the San Martín Tuxtla volcano (including *W. gladioliflora*, *W. nocturna* and *W. vanhyningii*; Espejo-Serna *et al.* 2005; Krömer *et al.* 2013). Together with chiropterophilous plants of other families, e.g. *Marcgravia mexicana* (Marcgraviaceae), *Mucuna argyrophylla* (Leguminosae) and *Solandra maxima* (Solanaceae) (CONANP 2006; P. A. Aguilar-Rodríguez, pers. obs.), these bromeliad species may provide nectar throughout the year as food for a local nectarivorous bat community, which comprises at least three species at the studied site (but see Coates *et al.* 2017). *Anoura geoffroyi* seems to be present in the area for most of the year, using the numerous caves for roosting. The presence of *G. soricina* was confirmed for 2–3 months of the year only, but this species could migrate to different altitudes (McGuire and Boyle 2013). In contrast, *H. underwoodi* is a rare species, the ecology of which is poorly known (Jones and Homan 1974), but it seems to prefer foraging in the canopy during the rainy season, and in the understory during the dry season (Samudio 2002), making it difficult to obtain reliable data for this species.

In conclusion, the studied bromeliads are self-compatible species that, at least three of them, used nectarivorous bats as pollinators. One of these bromeliads, *P.* recurvata, needs bat visitation to develop fruits, and accordingly, this species produces the best nectar reward in the habitat in comparison to the other bromeliads. Meanwhile, *W. nutans* seems to rely on self-pollination and cloning as a reproductive strategy, producing a poor nectar reward at individual plant level, compared to the other species. Taken all this into account, these bromeliads have developed different strategies to share (*W. noctiflorens*, *P. recurvata*) and partition (*P. viridiflora*, *P. recurvata*) nectar feeding bats as pollinators in the humid montane forest of San Martín Tuxtla volcano, therefore reducing the competition for its pollinators. These strategies include floral morphology, staggered flowering and differences in nectar quantity and quality. This study furthers our understanding of this highly adaptable plant family (Givnish *et al.* 2007) that possesses a remarkable variety of strategies with which to exploit its pollinators. Time of anthesis, floral morphology and nectar traits seem to be crucial characteristics to determine the main pollinators in Bromeliaceae, highlighting the importance to describe the natural history of the studied plant species, and the variations among sympatric species.

## Sources of Funding

This work was supported by the Consejo Nacional de Ciencia y Tecnología (CONACYT) (grant number 362134 awarded to P.A.A.-R.).

## Contributions by the Authors

P.A.A.-R., M.C.M.G., T.K. and J.G.G.-F. designed the research and experiments. P.A.A.-R. and M.C.M.G. conducted the field-work and analysed data. P.A.A.-R., M.C.M.G., T.K., J.G.G.-F. and M.T. wrote the manuscript.

## Conflict of Interest

None declared.

## Supporting Information

The following additional information is available in the online version of this article—

**Figure S1.** Flower of *Pitcairnia recurvata* with the stigma covered with a piece of drinking straw. After removal, the style and stigma showed no alteration, and returned to its natural position. In addition, the flower has been emasculated by removing the anthers.

**Table S1.** Data for the number of seeds per treatment and the nectar measurements, per bromeliad species.

**Video S1.** Bat visiting a flower of *Pitcairnia recurvata*, *ca.* 10 % of original speed.

**Video S2.** Bat visiting a flower of *Pseudalcantarea viridiflora*, *ca.* 10 % of original speed.

**Video S3.** Bat visiting a flower of *Werauhia noctiflorens*, *ca.* 10 % of original speed.

Supplementary Figure S1

Supplementary Tables S1

Supplementary Video S1

Supplementary Video S2

Supplementary Video S3
