# Supplementary material for: Bromeliads going batty: pollinator partitioning among sympatric chiropterophilous Bromeliaceae
Source: AoB Plants. 2019 Mar 12;11(2):plz014. doi: 10.1093/aobpla/plz014 (PMC6537948; doi:10.1093/aobpla/plz014)

**Figure S1** Flower of *Pitcairnia recurvata* with the stigma covered with a piece of drinking straw. After removal, the style and stigma showed no alteration, and returned to its natural position. In addition, the flower has been emasculated by removing the anthers.


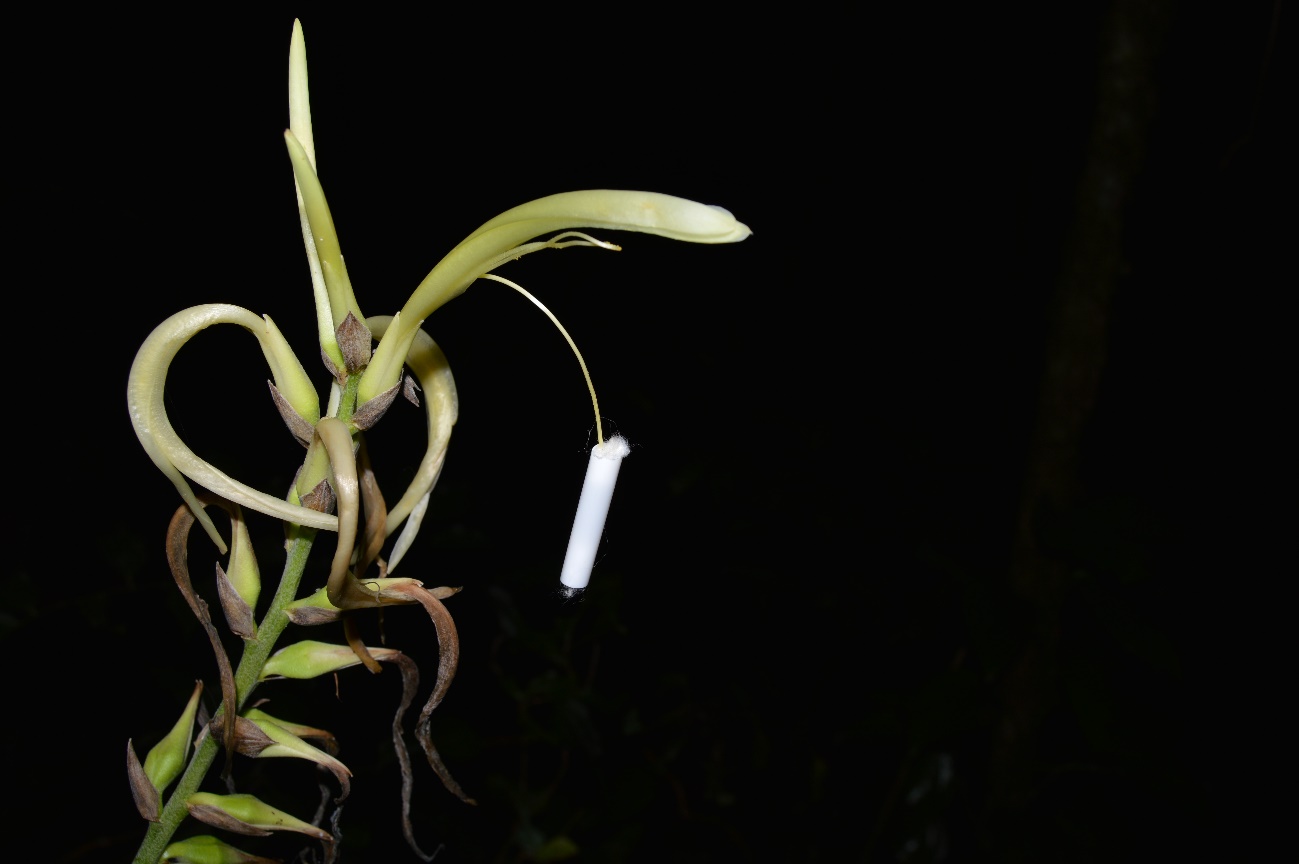

Supplement: Supplementary Figure S1 [file plz014_suppl_supplementary_figure_s1.docx]
